# Flavorful Microbial Fermentation: Metabolic, Flavoromic, and Sensory Analysis Insights Into Probiotic‐Enhanced Catharina Sour Beer With *Lacticaseibacillus paracasei* F19

**DOI:** 10.1155/ijm/3448995

**Published:** 2026-05-13

**Authors:** Marcos Edgar Herkenhoff, Ana Beatriz Praia, Guilherme Dilarri, Oliver Brödel, Marcus Frohme, Susana Marta Isay Saad

**Affiliations:** ^1^ Department of Biochemical and Pharmaceutical Technology, School of Pharmaceutical Sciences, University of São Paulo (USP), Av. Professor Lineu Prestes 580, São Paulo, 05508-000, São Paulo, Brazil, usp.br; ^2^ Food Research Center (FoRC), University of São Paulo (USP), Av. Professor Lineu Prestes 580, São Paulo, 05508-000, São Paulo, Brazil, usp.br; ^3^ Department of Fisheries Engineering and Biological Sciences, Santa Catarina State University (UDESC), Rua Coronel Fernandes Martins 270, Laguna, 88790-000, Santa Catarina, Brazil; ^4^ Multicentric Graduate Program in Biochemistry and Molecular Biology (PMBqBM), Santa Catarina State University (UDESC), Avenida Luiz de Camões 2090, Lages, 88520-000, Santa Catarina, Brazil; ^5^ Division Molecular Biotechnology and Functional Genomics, Technical University of Applied Sciences Wildau, Wildau, Germany, th-wildau.de

**Keywords:** craft beer, flavoromics, HS-SPME/GC–MS, metabolomics, PMA-qPCR, probiotics

## Abstract

This study investigated how fruit matrices and the probiotic strain *Lacticaseibacillus paracasei* subsp. *paracasei* F19 (F19) interact with *Saccharomyces cerevisiae* US‐05 to shape volatile formation, microbial viability, and sensory outcomes during the fermentation of Catharina sour beer. The central objective was to fill the current knowledge gap regarding dynamic flavor development in mixed fermentations, particularly in systems where probiotic LAB may contribute both functional and aromatic benefits. Microbial viability was monitored using propidium monoazide quantitative PCR (PMA‐qPCR); volatile compounds were profiled by headspace solid‐phase microextraction coupled to gas chromatography–mass spectrometry (HS‐SPME/GC–MS); and sensory acceptance was evaluated with 62 untrained panelists using a 9‐point hedonic scale. F19 displayed strong viability throughout fermentation (5–7 log CFU/mL), with significant increases after 58 days in the control and passion fruit formulations (*p* < 0.05), achieving the recommended daily intake level in 350‐mL servings. HS‐SPME/GC–MS identified 143 volatile compounds, including key aroma contributors, such as 3‐methylbutyl acetate, ethyl hexanoate, and ethyl octanoate, as well as 23 core volatiles persistent across all stages. Sensory evaluation revealed a clear preference for the passion fruit beer (6.94 ± 2.01), linked to 18 unique volatiles including 6‐methylhept‐5‐en‐2‐one and benzaldehyde, while the peach beer—despite containing honey‐like 2‐phenylacetaldehyde—showed lower acceptance (5.72 ± 1.91). Overall, this first integrated volatilomic, microbial, and sensory analysis demonstrates that *L. paracasei* F19 enhances aroma diversity and remains viable in fruit‐based sour beers, with passion fruit providing the most promising sensory and functional profile for innovative probiotic craft beer development.

## 1. Introduction

Traditional brewing methods have historically constrained beer style diversity and have not kept pace with contemporary consumer expectations for complex flavors and functional products [1–3]. Sour beers diverge from this model by introducing a lactic acid fermentation step before yeast‐driven alcoholic fermentation, resulting in a more acidic profile (pH 3.0–3.9) compared to conventional beers (pH 4.2) [4]. Several lactic acid bacteria are recognized as probiotics [5], and probiotics are defined as “live microorganisms which, when administered in adequate amounts, confer a health benefit on the host” [6]. This opens the possibility of using probiotic LAB to generate the acidic profile characteristic of Catharina sour while producing a beverage with potential functional benefits. Although beer is generally a hostile matrix for bacteria, recent studies confirm that Catharina sour can indeed be produced with probiotic strains [7, 8].

Sour beers can be produced through two main approaches known as kettle souring and mixed‐culture fermentation. In the kettle‐soured method, lactic acid bacteria are allowed to acidify the wort in the brewhouse kettle and are then inactivated by a second boil before yeast fermentation, a process considered rapid and highly controllable by commercial brewers [4, 9]. However, this second boil eliminates all LAB cells, resulting in beers without viable microorganisms, which limits both biochemical complexity and the possibility of incorporating live probiotic cultures. Mixed‐culture fermentation, however, allows LAB and yeast to act sequentially or simultaneously in the fermenter, supporting ongoing microbial activity and producing a broader spectrum of metabolic compounds associated with more layered sensory profiles [10–12]. From a probiotic science perspective, this method is more appropriate because LAB remain alive throughout the process and may contribute not only to acidification but also to the formation of aroma‐active compounds and potentially beneficial metabolic interactions.

Craft beer has gained strong traction among affluent consumer groups largely because its sensory attributes are both distinctive and rapidly embraced. These beers, produced from a wide array of raw materials, microbial strains, and innovative brewing approaches, exhibit complex matrices of macronutrients, micronutrients, and phytochemicals. Such diversity underscores the versatility of the craft beer sector and its capacity to offer a broad spectrum of flavors and nutritional elements. As the industry continues to innovate and expand, it consistently adapts to the preferences of an increasingly discerning consumer base, sustaining its dynamic momentum [13].

Building on these considerations, this study evaluated the volatile‐compound profile of Catharina sour beer produced with the probiotic strain *Lacticaseibacillus paracasei* F19 [14] in coculture with the top‐fermenting yeast *Saccharomyces cerevisiae* US‐05, a strain known for its clean fermentation profile and low diacetyl production at typical ale temperatures [15]. This coculture model provides a controlled framework for examining how microbial interactions shape the chemical and sensory development of sour beer.

**FIGURE 1:**
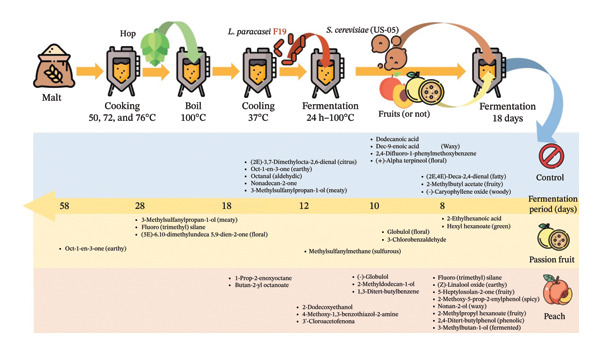
Flowchart of the execution of the work in the search for the fermentation metabolic pathway of the microorganism and its formed volatiles.

While previous studies have characterized aromatic markers of commercial beer styles using multivariate chemometrics [9], these analyses relied on finished and prefermented products, offering only static snapshots of volatile profiles. As a result, the biological and sensory transformations that occur during fermentation—especially in probiotic beer systems—remain largely unexplored. Early work by Praia et al. [7] demonstrated the technical feasibility of incorporating F19 into fruit‐based sour beers, but did not provide the mechanistic or integrative analyses needed to understand how microbial viability, volatile‐compound production, and sensory attributes evolve over time. To address these gaps, we examined how fruit matrices (passion fruit or peach) and the presence of the probiotic strain modulate the metabolism and production of volatile compounds throughout fermentation and maturation. Microbial survival was quantified by PMA‐qPCR; volatile compounds were monitored via headspace solid‐phase microextraction (HS‐SPME) coupled with gas chromatography–mass spectrometry (GC–MS); and sensory changes were assessed by trained panelists (Figure 1).

The novelty of this study lies in this integrated, time‐resolved analytical approach, which simultaneously tracks microbial viability, volatile‐compound dynamics, and sensory evolution in a probiotic‐enhanced Catharina sour beer. Rather than aiming to demonstrate probiotic efficacy—which would require clinical validation beyond the scope of this work—we focus on how *L. paracasei* F19 interacts with fruit substrates to reshape the flavoromic landscape along the full fermentation‐to‐maturation timeline. This multidimensional profiling captures both the succession of volatile metabolites and their sensory consequences, establishing a comprehensive framework for understanding flavor development in mixed‐culture fermentation systems. To our knowledge, this is the first study to integrate microbial, metabolomic, and sensory dimensions in the production of probiotic‐enhanced Catharina sour beer, providing methodological and conceptual advances relevant to craft and functional beverages.

## 2. Material and Methods

### 2.1. Cultures Employed

The recognized probiotic *Lacticaseibacillus paracasei* subsp. *paracasei* F19 (F19) (Chr. Hansen, Hørsholm, Denmark) was chosen for our beer formulations. This selection was based on the known capability and viability of this strain in alternative food matrices. This probiotic strain was employed in coculture with the yeast *S. cerevisiae* strain Safale US‐05 (US‐05) (Fermentis, Marcq en Baroeul, France), chosen for its previously highlighted attributes including its ability to produce a dry flavor in the beer [15], that is, without prominent volatile compounds, allowing the probiotic strain to have a more pronounced presence in the final flavor of the beer formulations. The strain, cryopreserved probiotic culture (maintained at −80°C in glycerol [800 μL of the probiotic strain in MRS broth and 200 μL of glycerol]) underwent activation at 37°C for 24 h using MRS broth (De Man‐Rogosa and Sharp, Oxoid, Thermo Fisher Scientific, Waltham, MA, USA) under anaerobic conditions (AnaeroGen™ Anaerobic System, Oxoid), following the protocol outlined by Battistini et al. [16]. The probiotic was thawed and then cultivated in MRS broth at 37°C for 24 h, twice consecutively. After cultivation, the cultures were centrifuged at 10000 × *g* for 10 min. The probiotic cells were then washed with sterile saline solution (0.85 g NaCl/100 mL) and homogenized using a stirrer. This washing process was repeated three times, and the supernatant was discarded each time. The final pellet obtained was resuspended and used to inoculate the beer formulations to achieve an approximate concentration of 8 log CFU/mL.

### 2.2. Production and Formulation of Catharina Sour Beer Wort and pH Determination During Fermentation

The Catharina sour formulations were designed by BeerSmith™ software, with the following characteristics: Standard Reference Method (SRM) 4, Original Gravity (OG) 1.045, Final Gravity (FG) 1.008, IBU 4, and Alcohol by Volume (ABV) 4.0%. Each 30 L batch of wort consisted of 4.38 kg of Pilsen malt and 4.38 kg of wheat malt, both from Castle Malting (Beloeil, Belgium), along with 25.2 g of Saaz hops (BarthHaas Group®, Nuremberg, Germany) with a 3.0% α‐acid content.

Each formulation was produced with 1 L in duplicate; to ensure consistency in the wort composition, a single batch of wort of 30 L was produced, including sensory analysis. The fermentation process involved three stages: (1) 50°C for 12 min; (2) 72°C for 60 min; and (3) 76°C for 10 min. After mashing, the wort underwent filtration and was boiled for 60 min with hops in a tank (Lamas Brew INOX 201, Lamas Brew, Campinas, Brazil). The filtered wort was cooled to 37°C using a chiller, transferred to a fermenter (Lamas Brew Shop, Campinas, SP, Brazil), inoculated with the *L. paracasei* F19 strain (8.0 log CFU/mL), and fermented at 37°C for 24 h under anaerobic conditions (AnaeroGen™ Anaerobic System, Oxoid). The pH reached 4.3 after the first fermentation at 24 h and ranged from 3.4 to 3.9 by the end of fermentation.

Next, the *S. cerevisiae* US‐05 strain (originally stored as a freeze‐dried yeast culture; Fermentis, Barœul, France) was first rehydrated in sterile water for 30 min and then added at 5.0 log CFU/mL, together with the fruit juice (passion fruit with apple [Mitto Sucos, Americana, São Paulo, Brazil; pH 3.2] or peach [Del Valle, Vallejo, Mexico City, Mexico; pH 4.0]). The alcoholic (second) fermentation was followed by over 8 days at 18°C and then maturation (2 days at 13°C, 3 days at 4°C, and 4 days at 0°C) and carbonation (11 days at 24°C). The beer was then bottled in sterilized glass bottles and stored at 4°C for 30 days. Using the Orion Three Stars equipment (Thermo Fisher Scientific, Waltham, Massachusetts, USA), with a penetration electrode, 2A04‐GF model (Analyser, São Paulo, SP, Brazil), the pH of the formulations was measured.

### 2.3. Propidium Monoazide Treatment and DNA Extraction

To prevent the amplification of DNA from deceased probiotic cells by quantitative PCR (qPCR), the beer samples were treated with propidium monoazide (PMA) (Biotium, Hayward, California, USA). To collect the probiotic cells, 1 mL of the beer sample was subjected to centrifugation (9000*g* for 10 min at 4°C) to form pellets, followed by two washes with Tris‐EDTA buffer (10 mM Tris‐HCL, 1 mM EDTA, pH 8). The pellets were then resuspended in 499 μL of phosphate‐buffered saline (PBS) before PMA treatment (Biotium). After suspension, 1.25 μL of PMA was introduced. Subsequently, the samples were incubated in the dark for PMA activation for 5 min and exposed, and for PMA deactivation, to LED light for 15 min using the Glo Plate™ Blue LED Illuminator (Biotium) [17].

DNA extractions were carried out using the MagMAX™ kit (Thermo Fisher Scientific) involving magnetic beads and the DynaMag™‐2 Magnet rack (Thermo Fisher Scientific, Waltham, Massachusetts, USA). The concentration, purity, and quality of the extracted nucleic acids were assessed using the NanoPhotometer® N60 spectrophotometer (Implen, Munich, Germany), and their integrity was verified through electrophoresis in a 1% agarose gel.

### 2.4. Viability of Microorganisms by PMA‐qPCR

The assessment of F19 and yeast viability was conducted through PMA‐qPCR, employing the ABI real‐time system 7500™ (Thermo Fisher Scientific) thermocycler. The amplification reactions included 12.5 μL of 2X Power SYBR®GreenPCR MasterMix (Applied Biosystems™), 5 μL of the DNA sample, and ultrapure water to reach a total volume of 25 μL.

For F19 (100 nM primer concentration), the cycling conditions were as follows: 1 cycle of 50°C for 2 min and 95°C for 10 min, followed by 40 cycles at 95°C for 15 s and 60°C for 30 s, with the primers F‐5′‐CGTGTGCCGATATAATGGGAACG‐3′ and R‐5′‐CCAAAGATCATCAAGCGTGCCAT‐3′, described by Sieuwerts and Håkansson [18]. For US‐05 (0.7 mM primer concentration), the cycling conditions were 1 cycle of 50°C for 2 min and 95°C for 10 min, and 40 cycles of 95°C for 15 s, 60°C for 1 min, and 72°C for 30 s, with the primers F‐5′‐GAAAACTCCACAGTGTGTTG‐3′ and R‐5′‐GCTTAAGTGCGCGGTCTTG‐3′, described by Zott et al. [19].

Standard curves were established using a 10‐fold dilution series of genomic DNA obtained from pure cultures of F19 (genome: 3,063,698 bp; GenBank: CP016355.1) and US‐05 (genome: 12,165,468 bp; GenBank: ASM308665v1), ranging from 100 to 1 × 10^8^ genome copies per amplification. The determination of viable cell equivalent numbers involved comparing the threshold cycle (Ct) of each sample with the standard curves [17].

### 2.5. PMA‐qPCR Statistical Analysis

For the statistical analysis, Minitab.ver19 software was employed, and the outcomes were presented as mean ± standard deviation (SD). To determine the quantity of microorganisms using PMA‐qPCR, Ct values were automatically transformed into CFU/mL by the 7500 Real‐Time PCR system software (Applied Biosystems™). Student’s *t*‐test was applied for comparing two samples, provided that a normal distribution was confirmed. In cases involving three or more samples, analysis of variance (ANOVA) was executed, followed by Tukey’s test. Significance in results was established when *p* ≤ 0.05 (5% significance level).

### 2.6. Metabolomic and Flavor Component Analysis by HS‐SPME/GC–MS

Metabolomic and flavoromic analyses were conducted utilizing a composite of the three formulations (control [without juice], with passion fruit juice, and peach juice) during the fermentation process, which included Days 1, 8, 10, 12, 18, 28, and 58 (*n* = 21). All analyses were performed in triplicate. The analytical procedures adhered to the methodology outlined by Giannetti et al. [20] and were refined following the enhancements proposed by Herkenhoff et al. [8]. To eliminate all CO_2_ from the samples, ensuring their suitability for analysis, the samples underwent degassing in an ultrasonic bath for 5 min at 5°C. Subsequently, 1.5 g of each sample was aliquoted into a 20‐mL autosampler headspace glass vial, and 0.8 g of sodium chloride was added to enhance extraction efficiency. The vials were safely sealed using a PTFE/silicone septum and a magnetic screw cap (Macherey‐Nagel, Bethlehem, PA, USA).

Profiles of volatile compounds were established through a HS‐SPME coupled with GC‐MS analysis. The GCMS‐QP2020 NX system equipped with a Nexis GC‐2030 gas chromatograph, a quadrupole mass spectrometer, and an AOC‐6000 Plus autosampler was employed (Shimadzu, Nakagyo‐ku, Kyoto, Japan). HS‐SPME extraction utilized a DVB/CAR/PDMS (divinylbenzene/carboxen/polydimethylsiloxane) Smart Fiber (80 μm) from Shimadzu.

Before analysis, the fiber underwent preconditioning at 240°C, and two blank injections were conducted, following the manufacturer’s instructions. The samples were equilibrated for 16 min at 60°C in the autosampler’s heat block. The extraction involved inserting the SPME fiber for 30 min into the headspace of the sample tubes. Subsequently, the fiber was introduced into the GC injector port for 3 min at 230°C in splitless mode (SPME glass liner, 0.75 mm ID), facilitating thermal desorption of volatile compounds. GC separation occurred under a consistent helium gas flow (1 mL/min) on a PEG (polyethylene glycol) capillary column (HP‐INNOWAX, 30 m, 0.25 mm i.d., 0.15 μm) from Shimadzu. The temperature program in the column oven comprised 40°C for 3 min, 5°C/min to 150°C, 15°C/min to 200°C, and a final hold at 200°C for 2 min.

Mass spectrometry detection, performed under electron impact (EI) ionization at 70 eV, operated in full‐scan acquisition mode in the 40–350 amu range. The transfer line and ion source were maintained at 250°C. Data acquisition utilized the total ion current (TIC) mode. Volatile compounds were identified by comparing the molecular fragmentation pattern of each peak with the mass spectra in the 2020 NIST MS database library (National Institute of Standards and Technology, Gaithersburg, USA). Compounds were considered identified when with a similarity index (SI) above 85. Uncertain identifications underwent confirmation through retention index calculation using a series of *n*‐alkanes (C8–C23) as a reference.

The identification of each volatile compound involved searching for the CAS Registry Number using the Yeast Metabolome Database (YMDB) [21] to assess their activities during fermentation and associations with yeast, and the Perflavory database (https://perflavory.com/search.php) was consulted for evaluating the flavor and aroma profiles of identified compounds. To quantify the volatile compounds shared by at least two of the analyzed beer styles, the detected peak areas were automatically converted into Area% using the LabSolutions GCMSsolution software (Shimadzu). This quantification strategy was chosen because, according to the manufacturer’s guidelines, employing a single standard would assume uniform concentrations across all samples for the remaining compounds. However, this assumption is not applicable due to the substantial variability among the beer styles analyzed.

### 2.7. Sensory Analysis

Sensory analyses were conducted with two distinct groups: one composed of students (*n* = 50) from the University of São Paulo (USP) (São Paulo, SP, Brazil) and another composed of students from the Escola Superior de Cerveja e Malte (ESCM) (*n* = 12) (Blumenau, SC, Brazil), thus a total of 62 individuals. The sensory acceptability assessment was conducted following a 30‐day refrigerated storage period at 4°C, utilizing a 9‐point hedonic scale [22]. In addition to evaluating the overall acceptability of the beverages, the purchase intention for each formulation was also appraised.

To ensure unbiased evaluations, the four distinct beer formulations were presented randomly to each participant, with each formulation undergoing assessment at least 50 times [23]. The participants comprised consumers (untrained volunteer tasters) aged between 25 and 65 years old, encompassing healthy adults of both genders. Before the evaluation of the beer formulations, volunteers carefully reviewed and signed a written consent form, confirming their willingness to participate in the sensory assessment.

In terms of inclusion criteria, the following were considered: Participants were healthy adults and regular consumers of craft beers. Exclusion criteria were as follows: Volunteers should not be pregnant; should not exhibit allergies or intolerances to any drink ingredients, including gluten or any other dietary restrictions (such as chronic illnesses or ongoing medical treatments with substances that may interact with the ingredients and/or impose restrictions on alcohol consumption); should abstain from driving vehicles for at least 60 min after the assessment; should not have a flu or cold; and should not have been exposed to strongly scented materials, food, or cosmetics before the sensory session.

Volunteers were presented with three samples, each containing 50 mL of a given formulation, in a monadic manner. The samples were served immediately after retrieval from the refrigerator, in clear plastic cups labeled with randomly assigned three‐digit codes. Participants were instructed to consume water between the evaluations of each offered sample. The sensory analysis was conducted following approval by the Research Ethics Committee of the School of Pharmaceutical Sciences at the USP (CAAE: 43047721.2.0000.0067).

## 3. Results and Discussion

### 3.1. pH Parameters and PMA‐qPCR Attested F19 Survival

**FIGURE 2:**
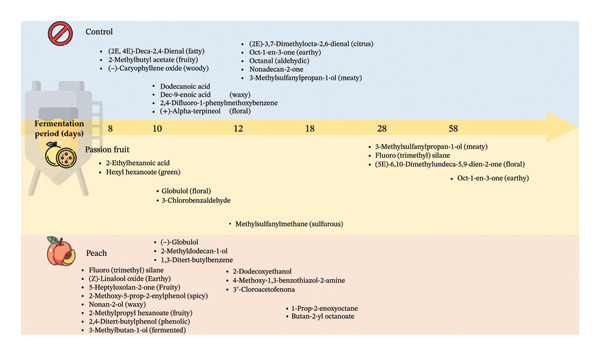
Scheme showing the production of unique compounds in each production period of the three formulations: control (without juice), with passion fruit juice, and with peach juice.

The results indicated the synthesis of distinct components throughout each manufacturing phase of the three formulations: control (without juice), with passion fruit juice, and with peach juice (Figure 2).

The pH of wort was 5.8. After the first fermentation, the pH was in the range of 4.35–4.57. At the end of the maturation period, all control (without fruit) groups showed pH between 3.7 and 3.8. Formulations with passion fruit and peach juice showed, respectively, pH 3.7 and 3.9 after maturation. The viability of F19 remained at a reasonable count throughout the fermentation process in all formulations. However, interestingly, at the end of 58 days, the population increased significantly in the control and passion fruit formulations compared to the 28‐day period (*p* < 0.05) (Table 1).

**TABLE 1:** Variations in pH, *Lacticaseibacillus paracasei* subsp. *paracasei* F19 (F19), and *Saccharomyces cerevisiae* strain US‐05 (US‐05) population (log equivalents) per 1 mL (CFU/mL) of Catharina sour beer by PMA‐qPCR throughout all production steps (Days 1–28) and after a month of storage (Day 58 at 4°C).

Formulation	Parameters	Period (days)						
		1	8	10	12	18	28	58
Control	F19 count	5.28 ± 0.02^A^	5.26 ± 0.03^A^	5.73 ± 0.04^A^	4.89 ± 0.03^A^	7.23 ± 0.04^A^	5.86 ± 0.04^A^	7.03 ± 0.02^A^
	US‐05 count	0	4.82 ± 0.04	5.49 ± 0.00	4.45 ± 0.00	7.19 ± 0.00	5.68 ± 0.01	7.17 ± 0.01
	pH	4.1 ± 0.2	3.9 ± 0.1	3.8 ± 0.1	3.8 ± 0.1	3.8 ± 0.1	3.8 ± 0.1	3.8 ± 0.1

Passion fruit	F19 count	5.28 ± 0.02^A^	6.30 ± 0.04^B^	6.09 ± 0.03^B^	4.60 ± 0.01^B^	4.82 ± 0.00^B^	5.38 ± 0.02^B^	7.02 ± 0.01^A^
	US‐05 count	0	5.31 ± 0.02	4.94 ± 0.03	3.5 ± 0.05	3.80 ± 0.03	5.44 ± 0.02	6.79 ± 0.02
	pH	3.8 ± 0.2	3.6 ± 0.1	3.6 ± 0.1	3.6 ± 0.2	3.6 ± 0.2	3.6 ± 0.2	3.6 ± 0.2

Peach	F19 count	5.28 ± 0.02^A^	6.36 ± 0.04^C^	5.72 ± 0.02^C^	4.84 ± 0.00^C^	5.10 ± 0.02^C^	5.81 ± 0.02^C^	5.20 ± 0.01^B^
	US‐05 count	0	5.22 ± 0.02	4.44 ± 0.03	3.87 ± 0.00	3.90 ± 0.02	5.11 ± 0.02	4.95 ± 0.01
	pH	4.4 ± 0.2	3.8 ± 0.2	3.8 ± 0.1	3.8 ± 0.1	3.8 ± 0.1	3.8 ± 0.1	3.8 ± 0.1

*Note:* All the formulations had the pH value of 5.8 ± 0.1 before the first fermentation.

^A–C^Different superscript capital letters in the same column denote significant differences among formulations for each time for F19 (*p* < 0.05).

Probiotic‐containing beer formulations showed viable counts consistent with potential probiotic beverages, particularly for those engaging in moderate or low beer consumption. The optimal daily probiotic intake remains a subject of ongoing debate in scientific investigations. In accordance with the guidelines established by the Canadian government [24], a food product must possess a minimum of 9 log CFU/mL of a recognized strain to be classified as a probiotic. Our study demonstrated the presence and viability of microorganisms at concentrations of 5 and 7 log CFU/mL, which are recognized as health‐promoting. Furthermore, this dose is within the limit considered as healthy for daily beer consumption, which would be equivalent to two 350 mL doses for men and one dose for women [25].

While the examined beer formulations incorporate probiotic strains, it is imperative to conduct *in vivo* studies and/or clinical trials to assess their probiotic potential comprehensively. Should future investigations provide evidence of favorable health effects, these beer formulations hold promise in terms of health functionality.

### 3.2. Classification by PLS‐DA

A supervised classification approach was designed to highlight differences attributable to the production process. The chromatographic profiles obtained from the GC‐MS TIC traces were examined through a PLS‐DA model, which allowed clear separation among the hop varieties under investigation (see Figure 3).

FIGURE 3Interpretative overview of the PLS‐DA model. In this panel, I relate the sample separation to the VIP scores and the associated regression coefficients, which together indicate the variables driving discrimination among production periods and formulations. (a) Day 1 in all formulations, (b) Day 8 in control formulation, (c) Day 8 in passion fruit formulation, (d) Day 8 in peach formulation, (e) Day 58 in control formulation, (f) Day 58 in passion fruit formulation, and (g) Day 58 in peach formulation. The highlighted chromatographic zones correspond to the TIC segments that most strongly influenced the multivariate model.

**Figure:**
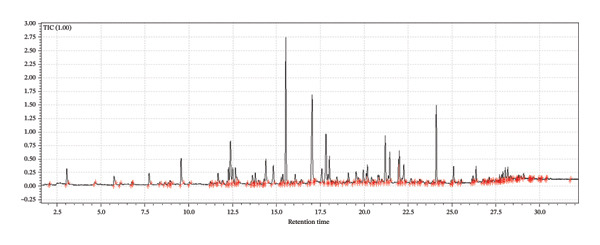
(a)

**Figure:**
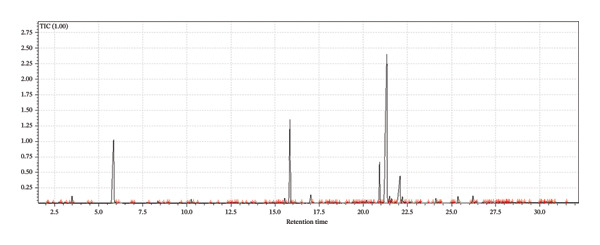
(b)

**Figure:**
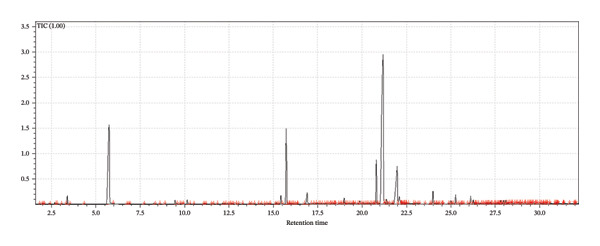
(c)

**Figure:**
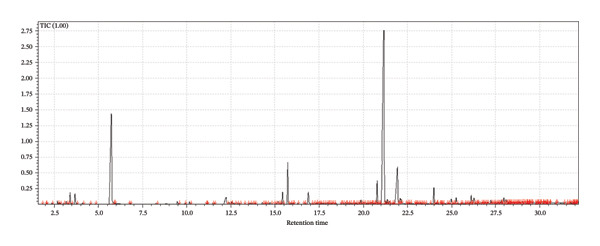
(d)

**Figure:**
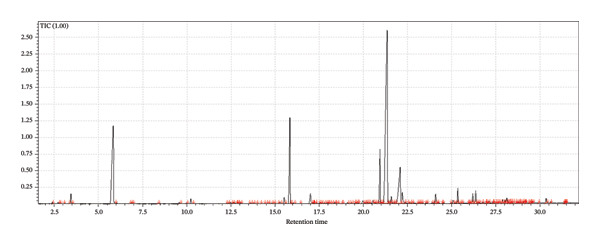
(e)

**Figure:**
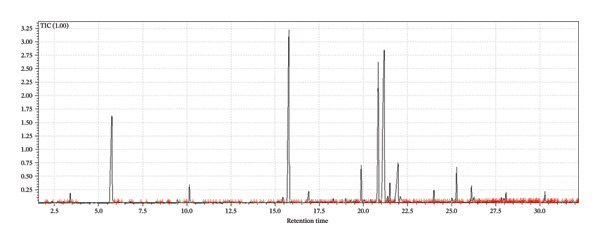
(f)

**Figure:**
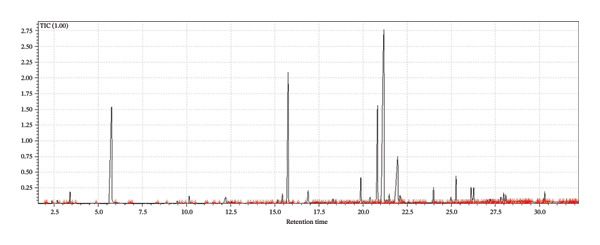
(g)

To explore the chemical distinctions in greater depth, I extracted the variable importance in projection (VIP) metrics generated by the PLS‐DA. These values describe how strongly each variable contributes to the model, with higher VIP scores indicating a greater role in discriminating the sample sets. Variables with normalized VIP values above 1 are generally interpreted as influential. By jointly evaluating the PLS regression weights and their corresponding VIP scores, it becomes possible to identify which compounds most strongly drive the separation among the groups and to infer the directionality of these compositional differences.

### 3.3. Volatile Aroma Development: Volatile Compounds Shared in All Beer Formulations

A total of 143 volatile compounds were identified by HS‐SPME/GC‐MS (Table 2). Of these, only 10 appeared exclusively on Day 1 of fermentation and disappeared thereafter, indicating early‐stage volatilization or rapid metabolic turnover. These day‐1‐specific compounds included ketones, aldehydes, terpenoid derivatives, and other low‐stability molecules, such as propan‐2‐one, (2E,4E)‐nona‐2,4‐dienal, and 2,4‐dimethylbenzaldehyde, while the complete list is provided in Table 2. In the first‐day GC‐MS results, all formulations were still identical because no fruit juice had yet been added; passion fruit and peach juices were incorporated only after the initial 24 h of lactic fermentation. The volatile profile observed on Day 1 therefore represents the metabolic activity of *Lacticaseibacillus paracasei* F19 alone, with no contribution from fruit‐derived substrates. Consequently, the compounds detected at this stage are common to the control, passion fruit, and peach formulations, as fruit‐specific volatiles could not yet emerge before the second fermentation.

**TABLE 2:** Compounds in *Lacticaseibacillus paracasei* subsp. *paracasei* F19 (F19) beer formulations without juice (control), with passion fruit juice, and with peach juice, identified by HS‐SPME/GC‐MS analysis.



*Note:* Compounds were identified in the Perflavory database to characterize odors and flavors. The filled field indicates the presence of the compound in different colors, with gray present in all formulations, blue present only in the control, yellow only in the formulation with passion fruit juice, light orange for peach juice, green for control and passion fruit, purple for control and peach, and dark orange for passion fruit and peach.

Sixteen compounds appeared only after the second day of fermentation—i.e., following yeast addition—and persisted until the end of beer production (3‐methyl‐4‐oxopentanoic acid; 3‐methylbutyl acetate; hexan‐1‐ol; ethyl hexanoate; ethyl octanoate; 2‐phenylethyl acetate; ethyl dec‐9‐enoate; 4‐ethenylphenol; decanoic acid; 5‐pentyloxolan‐2‐one; ethyl hexadecanoate; ethyl dodecanoate; ethyl propanoate; decan‐1‐ol; and diethyl butanedioate). Among these, 3‐methylbutyl acetate—derived from leucine metabolism—is valued for its banana‐like aroma in beer [26] and is also a relevant marker in olive oil [27]. Hexan‐1‐ol is widely used as a flavoring agent [28], imparting pleasant fruity notes at low concentrations but unpleasant aromas when excessive [29]. Ethyl hexanoate, produced through yeast‐mediated reactions between ethanol and fatty acids, contributes floral and fruity nuances, such as pineapple and green apple, and has a low odor threshold [30]. Ethyl octanoate similarly provides pear‐like fruity notes [31], and together, these esters play a central role in shaping wine aroma quality [32]. In contrast, 4‐ethenylphenol is associated with hospital‐ or plaster‐like off‐odors [33], and decanoic acid is recognized for its unpleasant fatty, soap‐like aroma ([34]; Perflavory database). Notably, none of these descriptors were reported by participants during sensory evaluation. Ethyl dodecanoate—responsible for sweet floral aromas in Neotinea orchids [35] and among the major esters in blueberry wine [36]—and ethyl propanoate, a common ester in the “Hongyu” mango lineage [37], also remained present throughout fermentation.

Furthermore, there are 23 compounds present in all formulations from prefermentation to its initiation and conclusion: undecan‐2‐one; nonanoic acid; 2‐ethylhexan‐1‐ol; 2‐methylbut‐3‐en‐2‐ol; 2‐methylbutyl; 2‐methylpropanoate; 3‐methylbutanoic acid; 2‐methylbutanoic acid; octan‐1‐ol; nonanal; 2‐phenylethanol; (1E,4E,8E)‐2,6,6,9‐tetramethylcycloundeca‐1,4,8‐triene; octanoic acid; humulenol‐II; tridecan‐2‐one; 4‐ethenyl‐2‐methoxyphenol; 3‐methylbutan‐1‐ol; 3,7‐dimethylocta‐1,6‐dien‐3‐ol; hexanoic acid; 1‐nonanol; and oct‐1‐en‐3‐ol (Table 2).

Undecan‐2‐one has been associated with warmed Welsh onion enriched with ascorbic acid, displaying a moderate fruity aroma and waxy taste [38]. Nonanoic acid, a precursor of nonanedioic acid via ω‐oxidation, contributes to the formation of this nine‐carbon dicarboxylic acid known for its antibacterial and anti‐inflammatory properties [39]. 2‐Methylbutyl acetate reinforces blackberry‐ and banana‐like notes and enhances fresh‐fruit and jam aromas even at subthreshold levels [40], whereas 3‐methylbutanoic acid is characterized by intense cheesy and sweaty impressions [41]. Nonanal, a widespread aldehyde in essential oils, such as cinnamon, rose, citrus, and pine, also exhibits antimicrobial and antifungal activity [42].

Octanoic acid, detected in Schwarzbier, is a versatile medium‐chain fatty acid and can be produced sustainably through microbial synthesis in *S. cerevisiae* [43]. It has further been proposed as a marker distinguishing industrial from craft beers [20]. The vinylphenol 4‐ethenyl‐2‐methoxyphenol, derived from ferulic acid through yeast‐mediated decarboxylation, forms pyranoanthocyanins relevant for wine color and imparts clove‐like notes in beer [33].

The oxylipin oct‐1‐en‐3‐ol, recognized for its earthy aroma, functions as a defense compound in marine algae [44–46] and occurs in crystal malt where it contributes to flavor complexity [47]. Its abundance varies in brown algae, such as *Cladostephus spongiosus* and *Halopteris scoparia* [48, 49], and is markedly reduced in dealcoholized beers [50], possibly influencing their negative sensory perception.

The aromatic alcohol 2‐phenylethanol, widely used in food, tobacco, cosmetics, and chemical products [51, 52] and also a substrate for ethyl phenylacetate synthesis [53], can be obtained either from plant extraction or microbial biotransformation [54]. Finally, tridecan‐2‐one, an essential oil constituent in several plant species, complements this set of compounds [55, 56].

### 3.4. Beers Do Not Taste the Same: Identification of Unique Volatile Compounds

By the end of beer production, no exclusive compounds were detected in the control group (Table 2), whereas two unique volatiles appeared in the peach formulation—2‐phenylacetaldehyde and 2,4,5‐trimethyl‐1,3‐dioxolane—and 18 in the passion fruit formulation, including 6‐methylhept‐5‐en‐2‐one, (4R)‐1‐methyl‐4‐prop‐1‐en‐2‐ylcyclohexene, (E)‐non‐2‐enal, benzaldehyde, styrene, undecan‐2‐ol, oct‐1‐en‐3‐one, 2‐methylpropyl hexanoate, and ethyl (Z)‐dec‐4‐enoate. Among the peach‐specific compounds, 2‐phenylacetaldehyde stands out for its strong green, honey‐like aroma and its role as an agonist of conserved plant volatile odorant receptors [57]. Its dose‐dependent activation of GmelOR4 and its status as a key amino acid–derived floral volatile are well documented across angiosperms [58–60]. The second peach‐specific compound, 2,4,5‐trimethyl‐1,3‐dioxolane, is known to cause off‐flavors in low‐alcohol wines produced by genetically modified *S. cerevisiae* through its spontaneous formation from acetaldehyde and 2,3‐butanediol [61], though no studies report its perception in beers.

Several of the passion fruit–specific volatiles also have clear biological or sensory relevance. The ketone 6‐methylhept‐5‐en‐2‐one occurs prominently in fresh samples of brown algae, such as *Cladostephus spongiosus* [48]. D‐limonene [(4R)‐1‐methyl‐4‐prop‐1‐en‐2‐ylcyclohexene], found in Okoume essential oil [62], shows antimicrobial activity through its action on cytoplasmic membranes [63]. Lipid‐oxidation derivatives, such as (E)‐non‐2‐enal, are characteristic of malt and particularly abundant in faba bean malt [41], while (E)‐oct‐2‐enal has been identified as a key flavor component in green wheat [64].

Benzaldehyde, another compound exclusive to the passion fruit formulation, is recognized for its almond‐like aroma and extensive use in food and pharmaceutical products [65]. Its bioactivity includes antioxidative and anti‐inflammatory effects well documented in Chinese herbal medicine, with reported benefits for headaches, dizziness, inflammatory processes, and even potential cognitive improvement [66, 67]. The food industry has also explored its enzymatic production from L‐phenylalanine [68], underscoring its technological relevance.

### 3.5. The Juice Matrix Influences the Synthesis of Aromatic Compounds

In interpreting the volatilomic patterns observed, it is important to consider the microbial and biochemical mechanisms underlying compound formation. During the first 24 h, when only *L. paracasei* F19 is active, the reduction in pH from 5.8 to approximately 4.3 (Table 1) reflects homofermentative hexose metabolism, a well‐established driver of acidification in sour beer systems [4]. This acidic shift contributes to the early appearance of short‐chain organic acids and higher alcohols. Once *S. cerevisiae* US‐05 is introduced together with the fruit matrix, ester formation increases substantially because yeast activity becomes dominant during alcoholic fermentation, consistent with cofermentation dynamics in mixed LAB‐yeast systems [69]. *S. cerevisiae* US‐05 is introduced together with the fruit matrix, and ester formation increases substantially because yeast alcohol acetyltransferases (AATs) catalyze the condensation between ethanol and medium‐chain fatty acids, giving rise to compounds, such as ethyl hexanoate and 3‐methylbutyl acetate [70]. These esters were especially intense between Days 10 and 28, coinciding with the peak yeast population in PMA‐qPCR.

In contrast, compounds that appear in all formulations throughout fermentation, including nonanal, octanoic acid, and 2‐phenylethanol, reflect shared metabolic pathways involving both microorganisms: Yeast‐driven Ehrlich catabolism [71] and lipid metabolism combine with LAB‐derived lactate and acetate fluxes to shape their temporal profile [4]. The viability patterns of F19 also help explain certain shifts: Its relative stability in the passion fruit formulation and decline in the peach formulation align with differences in pH and sugar utilization, factors known to modulate microbial performance in sour beer environments [4]. The viability patterns of F19 also help explain certain shifts: Its relative stability in the passion fruit formulation and decline in the peach formulation align with differences in pH and sugar utilization, which in turn affect the production of specific acids and alcohols through alcohol dehydrogenase activity and related oxidoreductase pathways [72]. Together, these microbial dynamics provide the mechanistic basis for the volatilomic evolution described below.

**FIGURE 4:**
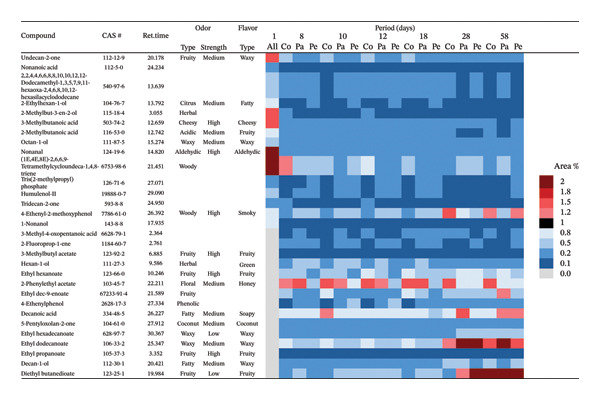
Heatmap showing the differential expression of compounds (area %) common to the three formulations: control (Co), with passion fruit juice (Pa), and with peach juice (Pe), across the production period.

Based on the experiments conducted, it is evident that each formulation expresses distinct temporal patterns of volatile synthesis, with several compounds emerging at specific stages of production (Figure 4). In the control formulation, 2‐methylbutyl acetate appeared on Day 8, a compound associated in the YMDB with alcohol O‐acetyltransferase, the enzyme catalyzing the esterification of isoamyl alcohol and other alcohols via acetyl‐CoA [21]. By Day 12, the same formulation also produced oct‐1‐en‐3‐one, a key odorant previously identified in the volatile fraction of steamed yeasted wheat dough [73].

In the passion fruit formulation, dimethyl sulfide (methylsulfonylmethane, DMS) was detected on Day 12. Although DMS is a well‐known off‐flavor, it dissipated in later stages, indicating a transient formation pattern. In contrast, the peach formulation exhibited the early synthesis of 4‐methoxy‐1,3‐benzothiazol‐2‐amine (propionic acid) on Day 12. This compound arises from microbial fermentation of glucose, glycerol, or lactic acid [74], and its biosynthesis proceeds via the acrylate pathway, in which the intermediate acryloyl‐CoA is toxic and becomes increasingly stable at higher pH. This pathway contributes to acetyl‐CoA production, a precursor for nuclear histone acetylation, and functions as the “anaerobic” isozyme of acetyl‐CoA synthetase, supporting growth on fermentable carbon sources and participating in the pyruvate dehydrogenase bypass [21]. Exposure of *S. cerevisiae* to subinhibitory concentrations of propionic acid has been shown to elevate endocytosis, alter cell cycle progression, and disrupt cellular respiration [75].

Although many compounds listed in the YMDB remain poorly characterized, the patterns observed here support the conclusion that the juice matrix—whether peach, passion fruit, or absent—modulates not only which volatiles are present, but also which compounds microorganisms synthesize during fermentation.

### 3.6. Volatile‐Compound Quantification and Clustering Reveal Treatment‐ and Time‐Dependent Aroma Profiles in the Formulations

Among the volatile compounds analyzed, fruity esters clearly emerged as the primary contributors to the sensory identity of the formulations (Figure 3). Ethyl hexanoate and ethyl octanoate were particularly abundant in both fruit‐based beers—passion fruit (Pa) and peach (Pe)—with concentrations peaking between Days 10 and 28 (Table 3), while remaining absent or minimal in the control (Co). These esters impart intense pineapple‐ and banana‐like aromas, making them highly desirable for enhancing the sensory value of fermented beverages. Ethyl acetate, although detected across all samples, accumulated most noticeably in the peach formulation, contributing sweet, fruity, and slightly solvent‐like notes that, in moderate concentrations, enrich aromatic complexity. The sensory impressions associated with *L. paracasei* fermentation products—largely floral and fruity—aligned well with the odor characteristics of the volatiles detected [76], a consequence of metabolic pathways that generate polyphenols and flavonoids [77]. Among these, flavonoids are notable for their antioxidant properties and their relevance to fermentation‐derived aroma formation [78]. Carbohydrate type also shaped volatile production: *L. paracasei*, being homofermentative, generates lactate from hexoses and lactate‐acetate mixtures from pentoses, while fructose and maltose metabolism may lead to elevated synthesis of volatiles and flavonoids, consistent with the GC‐MS patterns observed.

**TABLE 3:** Quantification of volatile compounds by area % identified in the three formulations: control (Co), with passion fruit juice (Pa), and with peach juice (Pe), across the production period.

Compound	CAS #	Ret. index	Odor		Flavor	Period (days)																		
			Type	Strength	Type	1	8			10			12			18			28			58		
						All	Co	Pa	Pe	Co	Pa	Pe	Co	Pa	Pe	Co	Pa	Pe	Co	Pa	Pe	Co	Pa	Pe
Propan‐2‐one	67‐64‐1	455	Solvent	High		0.070 ± 0.008																		
4‐Methylene‐methyl ester hexanoic acid	73805‐48‐8	951				1.084 ± 0.208																		
2‐Acetylthiazole	24295‐3‐2	1068				0.344 ± 0.127																		
(1S,5S,6R)‐2,6‐Dimethyl‐6‐(4‐methylpent‐3‐enyl)bicyclo[3.1.1]hept‐2‐ene	13474‐59‐4	1430				0.210 ± 0.143																		
(2E,4E)‐Nona‐2,4‐dienal	5910‐87‐2	1120	Fatty	High	Fatty	0.295 ± 0.155																		
(2Z)‐3,7‐Dimethylocta‐2,6‐dien‐1‐ol	106‐25‐2	1228	Floral	Medium	Citrus	0.580 ± 0.050																		
(3S,4aR,8aS)‐8a‐Methyl‐5‐methylidene‐3‐prop‐1‐en‐2‐yl‐1,2,3,4,4a,6,7,8‐octahydronaphthalene	17066‐67‐0	1469	Herbal	Medium		0.567 ± 0.221																		
2,4‐Dimethylbenzaldehyde	15764‐16‐6	1208	Naphthyl		Naphthyl	1.710 ± 0.010																		
2‐Methylbutyl 2‐methylpropanoate	2445‐69‐4	Fruity				0.227 ± 0.029																		
2‐Chlorobenzaldehyde	89‐98‐5	1162				0.925 ± 0.245			0.180 ± 0.001															
(Z)‐Oct‐2‐en‐1‐ol	26001‐58‐1	1067	Sweet floral			1.305 ± 0.205							0.140 ± 0.022			0.100 ± 0.020		0.100 ± 0.020						
2‐Pentylfuran	3777‐69‐3	1040	Fruity		Green	0.590 ± 0.259							0.090 ± 0.010											
(6E)‐7,11‐Dimethyl‐3‐methylidenedodeca‐1,6,10‐triene	18794‐84‐8	1440	Woody			1.323 ± 0.588							0.367 ± 0.020			0.257 ± 0.029				0.110 ± 0.001				
(Z)‐Undec‐6‐en‐2‐one	107853‐70‐3	1259				0.640 ± 0.056	0.050 ± 0.008						0.050 ± 0.008											
(2‐Chlorophenyl)methanol	17849‐38‐6	1216				0.257 ± 0.038				0.060 ± 0.010		0.085 ± 0.005					0.067 ± 0.020		0.067 ± 0.009	0.050 ± 0.008	0.060 ± 0.001	0.063 ± 0.005	0.053 ± 0.005	0.083 ± 0.012
Pentan‐1‐ol	71‐41‐0	761	Fermented		Fusel	0.233 ± 0.021	0.017 ± 0.005					0.017 ± 0.005							0.013 ± 0.005	0.013 ± 0.005				
Hexanal	66‐25‐1	806	Green	High	Green	0.337 ± 0.328	0.037 ± 0.005						0.053 ± 0.005			0.030 ± 0.001	0.030 ± 0.001						0.017 ± 0.005	
6‐Methylhept‐5‐en‐2‐one	110‐93‐0	938	Citrus	Medium	Green	1.660 ± 0.059							0.260 ± 0.016			0.173 ± 0.049		0.187 ± 0.045		0.157 ± 0.005			0.223 ± 0.005	
2‐Phenylacetaldehyde	122‐78‐1	1081	Green	High	Honey	3.637 ± 1.593	0.110 ± 0.010		0.110 ± 0.001															
(4R)‐1‐Methyl‐4‐prop‐1‐en‐2‐ylcyclohexene	5989‐27‐5	1018	Citrus	Medium	Citrus	0.380 ± 0.220	0.030 ± 0.001	0.027 ± 0.005					0.030 ± 0.008			0.017 ± 0.005		0.020 ± 0.001		0.023 ± 0.005			0.050 ± 0.008	
7‐Methyl‐3‐methylideneocta‐1,6‐diene	123‐35‐3	958	Spicy	High	Woody	1.800 ± 1.069	0.183 ± 0.020	0.040 ± 0.008		0.043 ± 0.005			0.177 ± 0.017			0.090 ± 0.014			0.010 ± 0.001	0.033 ± 0.009			0.053 ± 0.005	
(E)‐Non‐2‐enal	18829‐56‐6	1112	Fatty	High	Green	0.345 ± 0.145	0.060 ± 0.001		0.033 ± 0.009	0.035 ± 0.005			0.067 ± 0.012							0.030 ± 0.008			0.033 ± 0.005	
Heptanoic acid	111‐14‐8	1073	Cheesy		Waxy	1.117 ± 0.181	0.067 ± 0.017	0.009 ± 0.001		0.100 ± 0.021	0.137 ± 0.020	0.187 ± 0.053	0.095 ± 0.015	0.133 ± 0.069		0.127 ± 0.025	0.117 ± 0.020	0.147 ± 0.012						
(E)‐Oct‐2‐enal	2548‐87‐0	1013	Fatty	High	Fatty	1.043 ± 0.486	0.120 ± 0.008			0.037 ± 0.005			0.167 ± 0.020			0.090 ± 0.008				0.047 ± 0.009			0.067 ± 0.005	
(1R,4E,9S)‐4,11,11‐Trimethyl‐8‐methylidenebicyclo[7.2.0]undec‐4‐ene	87‐44‐5	1494	Spicy	Medium	Spicy	1.080 ± 0.569	0.317 ± 0.088	0.205 ± 0.005	0.163 ± 0.059	0.095 ± 0.005		0.180 ± 0.014	0.270 ± 0.030		0.120 ± 0.050					0.173 ± 0.058			0.237 ± 0.082	
2‐[(2R,4aR,8aR)‐4a,8‐Dimethyl‐2,3,4,5,6,8a‐hexahydro‐1H‐naphthalen‐2‐yl]propan‐2‐ol	473‐16‐5	1598				0.280 ± 0.028	0.055 ± 0.005	0.067 ± 0.017	0.073 ± 0.017	0.045 ± 0.005	0.097 ± 0.026	0.093 ± 0.026	0.050 ± 0.001	0.080 ± 0.016	0.083 ± 0.020	0.047 ± 0.017	0.073 ± 0.024	0.097 ± 0.026						
Benzaldehyde	100‐52‐7	982	Fruity	High	Fruity	2.347 ± 0.167	0.050 ± 0.014	0.067 ± 0.005	0.210 ± 0.001	0.035 ± 0.005	0.073 ± 0.005	0.233 ± 0.012	0.060 ± 0.016	0.063 ± 0.005	0.260 ± 0.029	0.043 ± 0.012	0.053 ± 0.024	0.247 ± 0.009		0.070 ± 0.016			0.050 ± 0.008	
Dodecan‐1‐ol	112‐53‐8	1457	Waxy	Medium	Soapy	1.375 ± 0.125	0.083 ± 0.026	0.450 ± 0.099	0.627 ± 0.049	0.277 ± 0.017	0.760 ± 0.102	0.687 ± 0.046	0.100 ± 0.001	0.787 ± 0.078	0.820 ± 0.110	0.157 ± 0.031	0.820 ± 0.070	0.727 ± 0.029	0.353 ± 0.009		0.240 ± 0.001	0.377 ± 0.045		0.567 ± 0.058
Undecan‐2‐one	112‐12‐9	1251	Fruity	Medium	Waxy	1.425 ± 0.065	0.440 ± 0.033	0.293 ± 0.048	0.205 ± 0.005	0.320 ± 0.029	0.230 ± 0.028	0.170 ± 0.010	0.447 ± 0.024	0.237 ± 0.026	0.160 ± 0.016	0.353 ± 0.026	0.303 ± 0.074	0.150 ± 0.030	0.170 ± 0.080	0.230 ± 0.040	0.177 ± 0.039	0.250 ± 0.010	0.200 ± 0.001	0.200 ± 0.001
Nonanoic acid	112‐5‐0	1272				0.215 ± 0.005	0.057 ± 0.019	0.090 ± 0.022	0.087 ± 0.009	0.053 ± 0.009	0.073 ± 0.031	0.080 ± 0.024	0.050 ± 0.001	0.063 ± 0.017	0.090 ± 0.024	0.057 ± 0.009	0.060 ± 0.001	0.090 ± 0.001	0.043 ± 0.005	0.060 ± 0.008	0.067 ± 0.005	0.047 ± 0.009	0.050 ± 0.008	0.076 ± 0.012
2‐Ethylhexan‐1‐ol	104‐76‐7	995	Citrus	Medium	Fatty	0.687 ± 0.221	0.040 ± 0.001	0.090 ± 0.001	0.117 ± 0.024	0.043 ± 0.005	0.087 ± 0.012	0.100 ± 0.001	0.035 ± 0.005	0.093 ± 0.005	0.110 ± 0.010	0.037 ± 0.009	0.100 ± 0.020	0.100 ± 0.010	0.060 ± 0.001	0.055 ± 0.005	0.055 ± 0.005	0.060 ± 0.001	0.070 ± 0.008	0.097 ± 0.005
2‐Methylbut‐3‐en‐2‐ol	115‐18‐4	600	Herbal			1.345 ± 0.075	0.140 ± 0.016	0.047 ± 0.012	0.047 ± 0.012	0.047 ± 0.005	0.030 ± 0.001	0.040 ± 0.008	0.167 ± 0.012	0.033 ± 0.005	0.030 ± 0.001	0.097 ± 0.005	0.030 ± 0.001	0.030 ± 0.001	0.033 ± 0.005	0.060 ± 0.001	0.057 ± 0.012	0.027 ± 0.005	0.087 ± 0.005	0.020 ± 0.001
3‐Methylbutanoic acid	503‐74‐2	811	Cheesy	High	Cheesy	1.350 ± 0.001	0.177 ± 0.012	0.147 ± 0.017	0.290 ± 0.024	0.173 ± 0.012	0.137 ± 0.005	0.277 ± 0.009	0.190 ± 0.022	0.143 ± 0.005	0.280 ± 0.008	0.170 ± 0.008	0.147 ± 0.017	0.287 ± 0.012	0.143 ± 0.005	0.083 ± 0.009	0.130 ± 0.008	0.140 ± 0.008	0.100 ± 0.001	0.197 ± 0.012
2‐Methylbutanoic acid	116‐53‐0	811	Acidic	Medium	Fruity	0.720 ± 0.010	0.147 ± 0.012	0.133 ± 0.025	0.163 ± 0.009	0.130 ± 0.008	0.127 ± 0.005	0.153 ± 0.012	0.127 ± 0.017	0.113 ± 0.024	0.167 ± 0.012	0.123 ± 0.005	0.130 ± 0.008	0.170 ± 0.014	0.110 ± 0.008	0.063 ± 0.005	0.077 ± 0.005	0.120 ± 0.014	0.093 ± 0.005	0.120 ± 0.001
Octan‐1‐ol	111‐87‐5	1059	Waxy	Medium	Waxy	0.483 ± 0.052	0.143 ± 0.005	0.217 ± 0.012	0.317 ± 0.024	0.137 ± 0.005	0.197 ± 0.005	0.320 ± 0.008	0.183 ± 0.017	0.203 ± 0.012	0.293 ± 0.033	0.130 ± 0.008	0.227 ± 0.082	0.317 ± 0.025	0.213 ± 0.009	0.190 ± 0.008	0.190 ± 0.008	0.207 ± 0.009	0.147 ± 0.005	0.320 ± 0.014
Nonanal	124‐19‐6	1104	Aldehydic	High	Aldehydic	2.210 ± 0.180	0.167 ± 0.029	0.193 ± 0.033	0.230 ± 0.020	0.137 ± 0.009	0.160 ± 0.008	0.207 ± 0.025	0.193 ± 0.012	0.157 ± 0.012	0.193 ± 0.005	0.137 ± 0.020	0.143 ± 0.005	0.200 ± 0.022	0.110 ± 0.001	0.117 ± 0.021	0.127 ± 0.005	0.100 ± 0.001	0.103 ± 0.009	0.127 ± 0.005
2‐Phenylethanol	60‐12‐8	1136	Floral	Medium	Floral	3.463 ± 0.299	37.400 ± 3.613	41.133 ± 1.851	43.083 ± 1.784	40.640 ± 2.324	40.817 ± 1.524	41.750 ± 1.890	34.977 ± 3.414	40.477 ± 1.283	41.670 ± 1.991	38.463 ± 2.546	38.980 ± 0.837	40.680 ± 1.431	40.907 ± 2.121	29.213 ± 2.634	28.750 ± 2.747	39.880 ± 2.552	25.797 ± 2.030	31.500 ± 1.223
(1E,4E,8E)‐2,6,6,9‐Tetramethylcycloundeca‐1,4,8‐triene	6753‐98‐6	1579	Woody			2.370 ± 0.010	1.103 ± 0.307	0.480 ± 0.042	0.545 ± 0.005	0.445 ± 0.025	0.250 ± 0.030	0.450 ± 0.040	0.945 ± 0.055	0.237 ± 0.026	0.307 ± 0.020	0.650 ± 0.059	0.305 ± 0.005	0.350 ± 0.033	0.290 ± 0.020	0.550 ± 0.040	0.590 ± 0.040	0.280 ± 0.001	0.397 ± 0.090	0.280 ± 0.008
Octanoic acid	124‐7‐2	1173				1.515 ± 0.075	7.750 ± 0.270	8.855 ± 0.035	8.290 ± 0.150	9.660 ± 0.030	10.653 ± 0.513	7.690 ± 0.300	7.955 ± 0.135	11.050 ± 0.463	8.243 ± 0.264	8.540 ± 0.030	10.620 ± 0.380	7.743 ± 0.255	8.825 ± 0.135	8.107 ± 0.040	5.715 ± 0.175	8.620 ± 0.100	7.550 ± 0.010	8.455 ± 0.195
Humulenol‐II	19888‐0‐7	1762				0.475 ± 0.015	0.060 ± 0.014	0.120 ± 0.010	0.165 ± 0.005	0.077 ± 0.012	0.190 ± 0.010	0.135 ± 0.025	0.085 ± 0.005	0.137 ± 0.029	0.205 ± 0.015	0.085 ± 0.005	0.155 ± 0.005	0.215 ± 0.005	0.067 ± 0.017	0.053 ± 0.009	0.053 ± 0.009	0.070 ± 0.014	0.053 ± 0.009	0.120 ± 0.029
Tridecan‐2‐one	593‐8‐8	1449				0.220 ± 0.010	0.110 ± 0.014	0.073 ± 0.009	0.073 ± 0.005	0.080 ± 0.008	0.070 ± 0.008	0.077 ± 0.005	0.113 ± 0.009	0.073 ± 0.005	0.073 ± 0.009	0.093 ± 0.005	0.080 ± 0.008	0.080 ± 0.001	0.063 ± 0.005	0.087 ± 0.012	0.090 ± 0.014	0.060 ± 0.008	0.083 ± 0.012	0.070 ± 0.001
4‐Ethenyl‐2‐methoxyphenol	7786‐61‐0	1293	Woody	High	Smoky	0.943 ± 0.253	0.255 ± 0.015	0.537 ± 0.070	0.635 ± 0.015	0.355 ± 0.015	0.555 ± 0.015	0.675 ± 0.015	0.340 ± 0.053	0.530 ± 0.030	0.625 ± 0.025	0.330 ± 0.053	0.565 ± 0.055	0.677 ± 0.054	1.300 ± 0.080	0.810 ± 0.050	0.483 ± 0.122	1.183 ± 0.098	0.505 ± 0.045	1.335 ± 0.005
3‐Methylbutan‐1‐ol	123‐51‐3	697	Fermented	High	Fusel	0.905 ± 0.015	14.573 ± 1.030	19.113 ± 1.258	19.340 ± 0.542	14.570 ± 0.873	19.137 ± 1.469	19.103 ± 1.253	15.405 ± 0.335	19.113 ± 0.606	18.687 ± 0.727	15.610 ± 0.390	18.330 ± 0.220	20.145 ± 0.075	15.653 ± 0.307	16.493 ± 0.768	14.720 ± 0.150	15.323 ± 0.785	14.403 ± 1.050	16.503 ± 0.980
3,7‐Dimethylocta‐1,6‐dien‐3‐ol	78‐70‐6	1082	Floral	Medium	Citrus	10.925 ± 0.635	0.657 ± 0.047	1.052 ± 0.005	1.240 ± 0.029	0.773 ± 0.052	1.130 ± 0.010	1.245 ± 0.005	0.820 ± 0.020	1.100 ± 0.073	1.250 ± 0.010	0.733 ± 0.049	1.040 ± 0.030	1.245 ± 0.025	0.690 ± 0.022	0.450 ± 0.024	0.377 ± 0.024	0.657 ± 0.009	0.453 ± 0.037	0.765 ± 0.005
Hexanoic acid	142‐62‐1	974	Fatty	Medium	Cheesy	8.935 ± 0.445	1.410 ± 0.001	2.035 ± 0.015	1.825 ± 0.015	1.475 ± 0.005	2.067 ± 0.086	1.705 ± 0.005	1.645 ± 0.005	2.127 ± 0.050	1.765 ± 0.005	1.625 ± 0.035	1.920 ± 0.020	1.675 ± 0.015	1.477 ± 0.034	1.270 ± 0.037	0.805 ± 0.005	1.425 ± 0.005	1.120 ± 0.001	1.360 ± 0.050
1‐Nonanol	143‐8‐8	1159				0.750 ± 0.010	0.077 ± 0.009	0.070 ± 0.008	0.090 ± 0.008	0.057 ± 0.005	0.060 ± 0.008	0.097 ± 0.005	0.103 ± 0.009	0.060 ± 0.008	0.100 ± 0.014	0.060 ± 0.008	0.057 ± 0.005	0.093 ± 0.005	0.057 ± 0.005	0.050 ± 0.008	0.080 ± 0.001	0.050 ± 0.008	0.060 ± 0.001	0.080 ± 0.008
Oct‐1‐en‐3‐ol	3391‐86‐4	969	Earthy	High	Mushroom	4.580 ± 0.270	0.333 ± 0.009	0.153 ± 0.019		0.150 ± 0.008	0.123 ± 0.017		0.430 ± 0.020	0.127 ± 0.017		0.300 ± 0.008	0.110 ± 0.001		0.127 ± 0.005	0.130 ± 0.001		0.113 ± 0.005	0.180 ± 0.008	
3‐Methyl‐4‐oxopentanoic acid	6628‐79‐1	1046					0.093 ± 0.009	0.100 ± 0.008	0.090 ± 0.001	0.087 ± 0.005	0.110 ± 0.008	0.100 ± 0.001	0.103 ± 0.009	0.110 ± 0.008	0.100 ± 0.008	0.120 ± 0.008	0.120 ± 0.008	0.107 ± 0.005	0.117 ± 0.005	0.107 ± 0.026	0.090 ± 0.001	0.123 ± 0.005	0.117 ± 0.019	0.143 ± 0.005
3‐Methylbutyl acetate	123‐92‐2	820	Fruity	High	Fruity		0.110 ± 0.008	0.080 ± 0.008	0.043 ± 0.005	0.103 ± 0.009	0.103 ± 0.009	0.060 ± 0.008	0.123 ± 0.009	0.110 ± 0.008	0.070 ± 0.014	0.120 ± 0.014	0.120 ± 0.008	0.067 ± 0.012	0.120 ± 0.001	0.080 ± 0.029	0.053 ± 0.005	0.120 ± 0.008	0.093 ± 0.012	0.087 ± 0.005
Hexan‐1‐ol	111‐27‐3	860	Herbal		Green		0.140 ± 0.008	0.553 ± 0.038	0.347 ± 0.017	0.157 ± 0.012	0.590 ± 0.010	0.350 ± 0.010	0.167 ± 0.012	0.557 ± 0.031	0.347 ± 0.026	0.157 ± 0.012	0.525 ± 0.035	0.330 ± 0.001	0.147 ± 0.005	0.273 ± 0.005	0.130 ± 0.008	0.147 ± 0.005	0.293 ± 0.020	0.230 ± 0.008
Ethyl hexanoate	123‐66‐0	984	Fruity	High	Fruity		0.565 ± 0.015	0.430 ± 0.067	0.270 ± 0.001	0.495 ± 0.005	0.390 ± 0.008	0.210 ± 0.010	0.870 ± 0.001	0.413 ± 0.024	0.253 ± 0.037	0.790 ± 0.020	0.440 ± 0.020	0.300 ± 0.033	0.495 ± 0.005	0.745 ± 0.005	0.990 ± 0.208	0.535 ± 0.005	1.060 ± 0.030	0.543 ± 0.065
Ethyl octanoate	106‐32‐1	1183	Waxy	Medium	Waxy		12.585 ± 0.145	5.205 ± 0.075	1.935 ± 0.085	10.780 ± 0.140	4.205 ± 0.305	2.510 ± 0.180	13.415 ± 0.115	4.365 ± 0.225	2.675 ± 0.165	12.570 ± 0.570	6.275 ± 0.995	4.333 ± 0.889	9.060 ± 0.240	14.457 ± 0.910	14.530 ± 0.550	9.610 ± 0.020	16.965 ± 0.115	10.540 ± 1.415
2‐Phenylethyl acetate	103‐45‐7	1259	Floral	Medium	Honey		1.085 ± 0.025	1.410 ± 0.040	0.610 ± 0.050	1.500 ± 0.010	1.330 ± 0.080	0.733 ± 0.111	1.275 ± 0.065	1.295 ± 0.005	0.595 ± 0.065	1.445 ± 0.095	1.300 ± 0.030	0.613 ± 0.086	1.567 ± 0.180	0.923 ± 0.188	0.755 ± 0.055	1.507 ± 0.090	0.850 ± 0.001	0.690 ± 0.030
Ethyl dec‐9‐enoate	67233‐91‐4	1371	Fruity				0.590 ± 0.060	0.105 ± 0.005	0.170 ± 0.075	0.785 ± 0.035	0.183 ± 0.042	0.220 ± 0.049	0.255 ± 0.025	0.223 ± 0.042	0.180 ± 0.030	0.710 ± 0.080	0.460 ± 0.202	0.273 ± 0.071	0.630 ± 0.107	0.303 ± 0.045	0.347 ± 0.034	0.683 ± 0.122	1.070 ± 0.107	0.507 ± 0.052
4‐Ethenylphenol	2628‐17‐3	1104	Phenolic				0.030 ± 0.008	0.183 ± 0.005	0.340 ± 0.036	0.043 ± 0.005	0.160 ± 0.010	0.377 ± 0.024	0.030 ± 0.008	0.183 ± 0.009	0.347 ± 0.021	0.037 ± 0.009	0.190 ± 0.010	0.333 ± 0.012	0.117 ± 0.012	0.137 ± 0.012	0.180 ± 0.016	0.120 ± 0.008	0.123 ± 0.012	0.263 ± 0.026
Decanoic acid	334‐48‐5	1372	Fatty	Medium	Soapy		0.980 ± 0.080	0.887 ± 0.065	0.803 ± 0.056	1.010 ± 0.010	0.985 ± 0.025	0.737 ± 0.049	0.933 ± 0.034	0.933 ± 0.034	0.710 ± 0.053	0.860 ± 0.086	0.903 ± 0.084	0.660 ± 0.037	0.920 ± 0.050	1.520 ± 0.060	0.785 ± 0.035	0.890 ± 0.050	1.090 ± 0.010	1.050 ± 0.040
5‐Pentyloxolan‐2‐one	104‐61‐0	1284	Coconut	Medium	Coconut		0.173 ± 0.005	0.233 ± 0.017	0.283 ± 0.017	0.223 ± 0.017	0.273 ± 0.039	0.320 ± 0.029	0.220 ± 0.008	0.297 ± 0.020	0.320 ± 0.029	0.223 ± 0.012	0.277 ± 0.048	0.330 ± 0.014	0.263 ± 0.019	0.233 ± 0.019	0.210 ± 0.008	0.270 ± 0.014	0.267 ± 0.009	0.340 ± 0.036
Ethyl hexadecanoate	628‐97‐7	1978	Waxy	Low	Waxy		0.130 ± 0.022	0.120 ± 0.045	0.123 ± 0.058	0.160 ± 0.016	0.173 ± 0.042	0.197 ± 0.019	0.133 ± 0.026	0.233 ± 0.026	0.217 ± 0.039	0.157 ± 0.037	0.210 ± 0.070	0.240 ± 0.075	0.287 ± 0.034	0.415 ± 0.025	0.505 ± 0.005	0.380 ± 0.022	0.615 ± 0.045	0.547 ± 0.049
Ethyl dodecanoate	106‐33‐2	1580	Waxy	Medium	Waxy		0.863 ± 0.168	0.490 ± 0.050	0.363 ± 0.150	0.795 ± 0.015	0.495 ± 0.045	0.350 ± 0.010	0.785 ± 0.055	0.535 ± 0.035	0.390 ± 0.030	0.695 ± 0.085	0.650 ± 0.030	0.460 ± 0.030	1.287 ± 0.143	2.310 ± 0.010	1.943 ± 0.290	1.407 ± 0.213	2.140 ± 0.010	1.580 ± 0.118
Ethyl propanoate	105‐37‐3	686	Fruity	High	Fruity		0.010 ± 0.001	0.030 ± 0.001	0.050 ± 0.008	0.010 ± 0.001	0.020 ± 0.001	0.050 ± 0.001	0.010 ± 0.001	0.020 ± 0.001	0.053 ± 0.005	0.010 ± 0.001	0.017 ± 0.005	0.053 ± 0.005	0.010 ± 0.001	0.017 ± 0.005	0.053 ± 0.005	0.010 ± 0.001	0.017 ± 0.005	0.073 ± 0.005
Decan‐1‐ol	112‐30‐1	1258	Fatty	Medium	Waxy		0.150 ± 0.008	0.203 ± 0.012	0.193 ± 0.005	0.160 ± 0.008	0.200 ± 0.016	0.217 ± 0.020	0.210 ± 0.001	0.207 ± 0.017	0.200 ± 0.001	0.150 ± 0.008	0.187 ± 0.020	0.197 ± 0.017	0.373 ± 0.029	0.347 ± 0.033	0.237 ± 0.017	0.377 ± 0.033	0.220 ± 0.008	0.473 ± 0.017
Diethyl butanedioate	123‐25‐1	1151	Fruity	Low	Fruity		0.185 ± 0.005	0.410 ± 0.030	0.333 ± 0.054	0.150 ± 0.001	0.353 ± 0.058	0.477 ± 0.074	0.377 ± 0.082	0.350 ± 0.067	0.477 ± 0.048	0.323 ± 0.052	0.360 ± 0.028	0.637 ± 0.058	0.340 ± 0.029	1.037 ± 0.125	3.170 ± 0.392	0.367 ± 0.034	2.460 ± 0.158	1.900 ± 0.119
Heptan‐1‐ol	111‐70‐6	960	Green	Medium	Solvent		0.140 ± 0.028	0.190 ± 0.001	0.267 ± 0.009	0.210 ± 0.014	0.190 ± 0.010	0.270 ± 0.001		0.133 ± 0.040	0.270 ± 0.010		0.150 ± 0.050		0.217 ± 0.005		0.203 ± 0.012	0.217 ± 0.005		0.203 ± 0.012
Ethyl heptanoate	106‐30‐9	1083	Fruity	Medium	Fruity		0.065 ± 0.005	0.037 ± 0.009		0.080 ± 0.028	0.030 ± 0.008	0.033 ± 0.012	0.130 ± 0.042	0.027 ± 0.005	0.034 ± 0.012	0.103 ± 0.026	0.053 ± 0.020	0.047 ± 0.009	0.070 ± 0.022	0.063 ± 0.009	0.130 ± 0.028	0.070 ± 0.022	0.113 ± 0.012	0.063 ± 0.012
1,1‐Diethoxyethane	105‐57‐7	705	Ethereal	High	Nutty		0.010 ± 0.001		0.170 ± 0.014	0.020 ± 0.001	0.010 ± 0.001	0.093 ± 0.012	0.020 ± 0.001	0.020 ± 0.001	0.103 ± 0.005	0.020 ± 0.001	0.013 ± 0.005	0.067 ± 0.005	0.017 ± 0.005	0.010 ± 0.001	0.010 ± 0.001	0.010 ± 0.001	0.010 ± 0.001	
3‐Methylbutyl octanoate	2035‐99‐6	1417	Fruity	Medium	Fruity		0.073 ± 0.026	0.040 ± 0.014		0.070 ± 0.028			0.083 ± 0.034	0.045 ± 0.015		0.070 ± 0.029	0.073 ± 0.020	0.040 ± 0.010	0.073 ± 0.020	0.100 ± 0.014	0.103 ± 0.009	0.087 ± 0.031	0.133 ± 0.031	0.093 ± 0.020
Propyl octanoate	624‐13‐5	1282	Coconut				0.027 ± 0.005			0.023 ± 0.005			0.023 ± 0.012			0.020 ± 0.001		0.020 ± 0.001	0.020 ± 0.001	0.023 ± 0.005	0.040 ± 0.008	0.017 ± 0.009	0.033 ± 0.005	0.033 ± 0.005
Styrene	100‐42‐5	883	Balsamic					0.230 ± 0.010	0.107 ± 0.009	0.170 ± 0.022	0.140 ± 0.022	0.140 ± 0.008	0.300 ± 0.040	0.130 ± 0.022	0.130 ± 0.020	0.240 ± 0.029	0.190 ± 0.057	0.175 ± 0.005		0.073 ± 0.025			0.117 ± 0.026	
2‐[(2R,5R)‐5‐Ethenyl‐5‐methyloxolan‐2‐yl]propan‐2‐ol	34995‐77‐2	1164	Floral	Medium				0.067 ± 0.005			0.073 ± 0.012		0.020 ± 0.001	0.063 ± 0.009		0.010 ± 0.001	0.045 ± 0.025		0.010 ± 0.001	0.035 ± 0.005	0.010 ± 0.001	0.025 ± 0.005	0.035 ± 0.005	0.015 ± 0.005
Undecan‐2‐ol	1653‐30‐1	1277	Waxy	Medium	Waxy		0.020 ± 0.001			0.015 ± 0.005	0.073 ± 0.012				0.010 ± 0.001	0.010 ± 0.001	0.045 ± 0.025	0.030 ± 0.001		0.035 ± 0.005			0.035 ± 0.005	
Phenylmethanol	100‐51‐6	1036	Floral	Medium	Fruity			0.100 ± 0.029	0.130 ± 0.024		0.105 ± 0.035	0.155 ± 0.005		0.075 ± 0.005	0.085 ± 0.065		0.140 ± 0.010			0.093 ± 0.012			0.117 ± 0.040	
Ethyl nonanoate	123‐29‐5	1282	Waxy	Medium	Waxy			0.100 ± 0.029		0.010 ± 0.001	0.105 ± 0.035	0.155 ± 0.005	0.010 ± 0.001	0.075 ± 0.005	0.085 ± 0.065	0.010 ± 0.001	0.140 ± 0.010	0.133 ± 0.025	0.010 ± 0.001	0.093 ± 0.012	0.137 ± 0.012	0.010 ± 0.001	0.117 ± 0.040	0.180 ± 0.016
2‐[(1S)‐4‐Methylcyclohex‐3‐en‐1‐yl]propan‐2‐ol	10482‐56‐1	1143	Terpenic					0.100 ± 0.029	0.130 ± 0.024		0.105 ± 0.035	0.155 ± 0.005		0.075 ± 0.005	0.085 ± 0.065		0.140 ± 0.010	0.133 ± 0.024	0.010 ± 0.001	0.093 ± 0.012	0.137 ± 0.012	0.010 ± 0.001	0.117 ± 0.040	0.180 ± 0.016
Ethyl ester decanoic acid	110‐38‐3	1381	Waxy	Medium	Waxy		6.230 ± 1.249	3.433 ± 0.607	1.387 ± 0.492	4.640 ± 0.773	2.883 ± 0.444	1.803 ± 0.364	6.403 ± 1.330	2.770 ± 0.299	1.820 ± 0.361		3.733 ± 0.555	2.310 ± 0.382				6.103 ± 0.959	10.917 ± 1.305	6.877 ± 0.529
2‐Nonyloxirane	17322‐97‐3	1205						0.203 ± 0.005	0.175 ± 0.005	0.117 ± 0.020	0.197 ± 0.020	0.125 ± 0.005			0.200 ± 0.010				0.073 ± 0.012	0.087 ± 0.019	0.080 ± 0.001	0.067 ± 0.012	0.080 ± 0.008	0.080 ± 0.001
(2E,4E)‐deca‐2,4‐dienal	25152‐84‐5	1220	Fatty	High	Fatty		0.030 ± 0.001						0.040 ± 0.001											
2‐Methylbutyl acetate	624‐41‐9	820	Fruity		Fruity		0.030 ± 0.001			0.030 ± 0.010						0.027 ± 0.005	0.027 ± 0.005	0.027 ± 0.005						
(1R,4R,6R,10S)‐4,12,12‐Trimethyl‐9‐methylidene‐5‐oxatricyclo[8.2.0.04,6]dodecane	1139‐30‐6	1507	Woody	Medium	Woody		0.063 ± 0.005			0.060 ± 0.022			0.080 ± 0.014			0.083 ± 0.026		0.060 ± 0.010						
4‐Chlorobenzaldehyde	104‐88‐1	1162					0.080 ± 0.001																	
Dec‐9‐enoic acid	14436‐32‐9	1362	Waxy	Medium	Waxy					0.153 ± 0.017														
2,4‐Difluoro‐1‐phenylmethoxybenzene	152434‐86‐1	1492								0.030 ± 0.001			0.050 ± 0.001											
2‐[(1R)‐4‐Methylcyclohex‐3‐en‐1‐yl]propan‐2‐ol	7785‐53‐7	1143	Floral	Medium						0.120 ± 0.001						0.117 ± 0.005		0.230 ± 0.001						
(2E)‐3,7‐Dimethylocta‐2,6‐dienal	141‐27‐5	1174	Citrus	Medium	Citrus								0.075 ± 0.055											
Oct‐1‐en‐3‐one	4312‐99‐6	943	Earthy	High	Earthy								0.105 ± 0.005										0.065 ± 0.005	
Octanal	124‐13‐0	1005	Aldehydic	High	Aldehydic								0.0637 ± 0.005											
Nonadecan‐2‐one	629‐66‐3	2046											0.115 ± 0.005											
3‐Methylsulfanylpropan‐1‐ol	505‐10‐2	912	Meaty		Onion								0.010 ± 0.001							0.077 ± 0.017				
(4‐Chlorophenyl)methanol	873‐76‐7	1216					0.037 ± 0.005	0.070 ± 0.010			0.070 ± 0.010		0.050 ± 0.010		0.090 ± 0.008	0.060 ± 0.008		0.080 ± 0.010						
2‐Ethylhexanoic acid	149‐57‐5	1109						0.057 ± 0.005						0.047 ± 0.005	0.030 ± 0.014									
Hexyl hexanoate	6378‐65‐0	1381	Green		Fruity			0.080 ± 0.001						0.050 ± 0.008			0.050 ± 0.001							
Globulol	51371‐47‐2	1530	Floral								0.050 ± 0.001													
3‐Chlorobenzaldehyde	587‐4‐2	1162									0.145 ± 0.015													
Methylsulfonylmethane	75‐18‐3	471	Sulfurous	High	Sulfurous									0.005 ± 0.001										
Propyl propanoate	106‐36‐5	785	Chemical		Tropical									0.030 ± 0.001	0.125 ± 0.005									
Fluoro(trimethyl)silane	420‐56‐4	202							0.060 ± 0.001											0.020 ± 0.014				
(1R,3Z,7Z,11R)‐1,5,5,8‐Tetramethyl‐12‐oxabicyclo[9.1.0]dodeca‐3,7‐diene	19888‐34‐7	1592							0.397 ± 0.069															
2,2‐Dimethylpropyl acetate	926‐41‐0	800							0.185 ± 0.005			0.175 ± 0.105												
2‐[(2R,5S)‐5‐Ethenyl‐5‐methyloxolan‐2‐yl]propan‐2‐ol	5989‐33‐3	1164	Earthy	Medium					0.025 ± 0.005			0.035 ± 0.005												
5‐Heptyloxolan‐2‐one	104‐67‐6	1483	Fruity	Medium	Creamy				0.080 ± 0.001			0.080 ± 0.001			0.080 ± 0.001									
2,6‐Ditert‐butyl‐4‐hydroxy‐4‐methylcyclohexa‐2,5‐dien‐1‐one	10396‐80‐2	1673							0.067 ± 0.005			0.087 ± 0.009			0.097 ± 0.005									
(4‐Hydroxyphenyl)phosphonic acid	33795‐18‐5	0							0.145 ± 0.005			0.155 ± 0.015			0.150 ± 0.010									
2‐Methoxy‐5‐prop‐2‐enylphenol	501‐19‐9	1392	Spicy						0.267 ± 0.009			0.305 ± 0.015			0.310 ± 0.001									
Tridecan‐3‐one	1534‐26‐5	1449							0.030 ± 0.001		0.030 ± 0.008	0.030 ± 0.008		0.023 ± 0.005	0.033 ± 0.005									
Nonan‐2‐ol	628‐99‐9	1078	Waxy		Waxy				0.025 ± 0.015			0.040 ± 0.020	0.020 ± 0.008											
2,4,5‐Trimethyl‐1,3‐dioxolane	3299‐32‐9	761							0.155 ± 0.015			0.133 ± 0.033			0.160 ± 0.042									0.010 ± 0.001
2‐Methylpropyl hexanoate	105‐79‐3	1118	Fruity	Medium	Fruity				0.010 ± 0.001			0.010 ± 0.001											0.020 ± 0.001	
2,4‐Ditert‐butylphenol	96‐76‐4	1555	Phenolic						0.380 ± 0.099		0.377 ± 0.106	0.430 ± 0.061		0.427 ± 0.088	0.530 ± 0.070	0.190 ± 0.010	0.190 ± 0.040	0.120 ± 0.001	0.270 ± 0.029					
3‐Methylbutan‐1‐ol	123‐51‐3	697	Fermented	Medium	Fusel			19.113 ± 1.258	19.340 ± 0.542		19.137 ± 1.469									16.493 ± 0.768	15.313 ± 0.848	15.323 ± 0.785	14.403 ± 1.050	16.503 ± 0.980
(1aR,4R,4aR,7R,7aS,7bS)‐1,1,4,7‐Tetramethyl‐2,3,4a,5,6,7,7a,7b‐octahydro‐1aH‐cyclopropa[e]azulen‐4‐ol	489‐41‐8	1530										0.060 ± 0.008												
2‐Methyldodecan‐1‐ol	57289‐26‐6	1492										0.045 ± 0.005												
1,3‐Ditert‐butylbenzene	1014‐60‐4	1334										0.085 ± 0.005												
2‐Dodecoxyethanol	4536‐30‐5	1731													0.123 ± 0.049									
4‐Methoxy‐1,3‐benzothiazol‐2‐amine	79‐9‐4	676													1.065 ± 0.035									
2,3,5,6‐Tetrachlorocyclohexa‐2,5‐diene‐1,4‐dione	99‐2‐5	1209													0.085 ± 0.025									
1‐Prop‐2‐enoxyoctane	3295‐97‐4	1181																0.010 ± 0.001						
Butan‐2‐yl octanoate	5458‐61‐7	1317																0.010 ± 0.001						
(Z)‐Non‐2‐enal	60784‐31‐8	1112	Fatty	High												0.050 ± 0.010		0.050 ± 0.010						
(4aS,8aR)‐8a‐Methyl‐4‐methylidene‐6‐propan‐2‐ylidene‐2,3,4a,5,7,8‐hexahydro‐1H‐naphthalene	515‐17‐3	1502	Woody	Medium												0.090 ± 0.010		0.090 ± 0.010						
2‐Methylpropyl acetate	110‐19‐0	721	Fruity	Medium	Fruity											0.010 ± 0.001		0.010 ± 0.001	0.015 ± 0.005					
(6E)‐3,7,11‐Trimethyldodeca‐1,6,10‐trien‐3‐ol	40716‐66‐3	1564	Floral	Low	Green																0.010 ± 0.001	0.020 ± 0.001		0.047 ± 0.019
Ethyl 2‐hydroxy‐3‐phenylpropanoate	15399‐5‐0	1521																				0.065 ± 0.005	0.130 ± 0.001	
2,2‐Dimethylpropyl acetate	926‐41‐0	800															0.020 ± 0.001			0.043 ± 0.020				
(5E)‐6,10‐Dimethylundeca‐5,9‐dien‐2‐one	3796‐70‐1	1420	Floral	Medium	Floral								0.090 ± 0.010							0.018 ± 0.001			0.360 ± 0.001	
Ethyl (Z)‐dec‐4‐enoate	7367‐84‐2	1389																					0.080 ± 0.016	
Ethyl 3‐Hydroxyoctanoate	7367‐90‐0	1345	Fruity																		0.020 ± 0.001	0.013 ± 0.005	0.017 ± 0.005	0.023 ± 0.005
n‐Caprylic acid isobutyl ester	03/06/5461	1317																	0.103 ± 0.029		0.135 ± 0.035	0.113 ± 0.026	0.157 ± 0.029	0.110 ± 0.022
3‐Methylbutyl decanoate	40716‐66‐3	1564	Waxy	Medium	Waxy															0.010 ± 0.001	0.010 ± 0.001	0.020 ± 0.001	0.010 ± 0.001	0.047 ± 0.019
Ethyl dl‐2‐hydroxycaproate	6946‐90‐3	1146																		0.013 ± 0.005	0.013 ± 0.005	0.013 ± 0.005	0.020 ± 0.001	0.010 ± 0.001
Ethyl 2‐phenylacetate	101‐97‐3	1259	Floral	High	Honey											0.030 ± 0.010		0.020 ± 0.001	0.057 ± 0.009	0.063 ± 0.012	0.100 ± 0.016	0.053 ± 0.009	0.120 ± 0.008	0.083 ± 0.012
Ethyl benzoate	93‐89‐0	1160	Minty	Medium	Medicinal										0.023 ± 0.012			0.030 ± 0.001	0.020 ± 0.008	0.043 ± 0.009	0.047 ± 0.005	0.017 ± 0.005	0.053 ± 0.005	0.047 ± 0.012
Ethyl ester tetradecanoic acid	124‐6‐1	1779					0.197 ± 0.062												0.367 ± 0.066	0.050 ± 0.024	0.527 ± 0.083	0.407 ± 0.012	0.513 ± 0.038	0.543 ± 0.037
Tetradecane	629‐59‐4	1400	Mild waxy	Low			0.037 ± 0.005						0.040 ± 0.010			0.035 ± 0.005	0.040 ± 0.014	0.030 ± 0.001		0.023 ± 0.009	0.040 ± 0.001	0.020 ± 0.001	0.033 ± 0.005	0.015 ± 0.005
2‐Methylpropyl decanoate	30673‐38‐2	1516	Fermented																0.040 ± 0.020	0.030 ± 0.008	0.030 ± 0.001	0.027 ± 0.009	0.047 ± 0.012	0.025 ± 0.005
Ethyl (E)‐hexadec‐9‐enoate	54546‐22‐4	1986																	0.053 ± 0.005	0.057 ± 0.005	0.077 ± 0.017	0.070 ± 0.008	0.087 ± 0.005	0.080 ± 0.001

Higher alcohols, such as isoamyl alcohol and 2‐phenylethanol, complemented the ester profile and were present in all formulations, though slightly more stable in the control. Notably, 2‐phenylethanol increased markedly in the passion fruit formulation, reinforcing its floral, rose‐like aroma. While these alcohols contribute to aromatic depth, excessive levels can become pungent, underscoring the need for balanced concentrations to preserve sensory quality.

Certain volatiles were formulation‐specific, strengthening the aromatic signatures of each beer. Hexyl acetate—linked to fresh apple‐ and pear‐like aromas—was characteristic of the peach formulation, increasing from Day 10 to Day 28. Linalool and geraniol, with floral and citrus nuances, were more abundant in the passion fruit formulation, while diacetyl (butanedione), with its buttery aroma, appeared more prominently in the control during early fermentation.

These chemical patterns translated into distinct sensory trajectories. The control formulation exhibited a simpler, more alcohol‐forward profile, lacking the aromatic richness imparted by fruit‐derived or fruit‐enhanced volatiles. In contrast, the passion fruit formulation presented a complex, tropical character driven by esters and floral compounds, especially between Days 10 and 28, making it the most aromatic and expressive of the three. The peach formulation displayed a softer fruit profile, dominated by hexyl acetate and ethyl octanoate, appealing to palates that favor milder, more delicate beverages.

Multivariate analysis deepened these distinctions by revealing clusters of volatiles with shared metabolic origins. Compounds, such as 3‐methylbutanoic acid, 3‐methylbutyl acetate, and ethyl hexanoate, are grouped together, suggesting derivation from short‐chain amino acid metabolism. A second cluster—including nonanal, decanoic acid, and undecan‐2‐one—aligned with lipid‐oxidation pathways, highlighting multiple biochemical routes contributing to volatilomic diversity across samples.

The effects of fermentation time and treatment further clarified volatile dynamics. Passion fruit and peach formulations at Days 28 and 58 exhibited an overall increase in volatile abundance, reflecting enhanced compound formation or accumulation in the presence of fruit matrices. Conversely, the control showed consistently lower concentrations, indicating limited chemical and microbiological transformation.

Several volatiles also acted as temporal markers. Early‐peaking compounds, such as 2‐methylbutanoic acid, may indicate initial fermentation, while later peaking volatiles, such as 4‐ethylphenol and ethyl dec‐9‐enoate, appear associated with maturation processes. These time‐dependent patterns reinforce the value of specific volatiles as biomarkers for monitoring fermentation progression and product quality.

Heatmap and cluster analyses strengthened these interpretations, illustrating how fermentation time and juice type jointly structure volatilomic outcomes. Such insights offer practical applications, including traceability through chemical markers, optimization of sensory attributes, and development of targeted quality control strategies for fruit‐based fermentations.

Finally, the compounds showing the greatest variability—tridecan‐2‐one (0.339), 4‐ethylphenol (0.337), 5‐pentyloxolan‐2‐one (0.336), octan‐1‐ol (0.332), 3‐methyl‐4‐octen‐3‐ol (0.321), 1‐nonanol (0.303), tris(2‐methylpropyl)phosphate (0.302), decanoic acid (0.295), and ethyl hexadecanoate (0.289)—displayed the widest fluctuations across treatments and time points, marking them as promising candidates for future biomarker development in fermentation monitoring, sensory prediction, and quality control.

### 3.7. Sensory Analysis and Its Relationship With HS‐SPME/GC‐MS

The sensory analyses revealed an evaluation of 5.94 (±1.82) by the USP group, corresponding to a rating ranging from “Slightly liked” to “Neither liked nor disliked.” However, there was a higher acceptance among the ESCM group, with a score of 6.58 (±0.76), equivalent to a rating ranging from “*Liked moderately*” to “*Slightly liked*.” These analyses also indicated a preference for the passion fruit beer samples among the USP group, with an acceptance of 6.94 (±2.01), corresponding to a rating between “Liked moderately” and “Slightly liked.” In contrast, the ESCM group rated it at 5.17 (±1.91), which corresponds to “Slightly liked” to “Neither liked nor disliked.” Regarding the peach samples, the USP group provided an evaluation of 5.72 (±1.91), and the ESCM group rated it at 5.00 (±1.89), indicating a rating of “Neither liked nor disliked.”

It is important to emphasize that in the sensory analysis evaluation form, a field for observations was provided so that volunteers could share their perceptions. For the passion fruit samples, especially among the USP group, several volunteers reported perceptions, such as fruity and citrus. In addition to the presence of passion fruit juice, HS‐SPME/GC‐MS analyses identified compounds related to these flavors and odors, such as 6‐methylhept‐5‐en‐2‐one (citrus), (4R)‐1‐methyl‐4‐prop‐1‐en‐2‐ylcyclohexene (citrus), benzaldehyde (fruity), and 2‐methylpropyl hexanoate (fruity).

Meanwhile, for the peach samples, some participants reported a sweet taste reminiscent of sugarcane or honey. According to HS‐SPME/GC‐MS analyses, 2‐phenylacetaldehyde was identified, which is described as having a honey‐like flavor.

## 4. Conclusions

The *Lacticaseibacillus paracasei* subsp. *paracasei* F19 (F19) remained consistently (5–7 log CFU/mL) throughout the fermentation. To date, this is the first study that involves the measurement of these compounds during the fermentation process in 3 formulations of Catharina sour. Compounds generated during fermentation, such as 3‐methylbutyl acetate (banana aroma), hexan‐1‐ol (fruity notes), ethyl hexanoate, and ethyl octanoate (fruity aromas), underscore the importance of the F19 probiotic strain in shaping the beer’s flavor. Exclusive volatile compounds related to beer in each formulation demonstrate that the fruit influences the final fermentation flavor. This study significantly enhances the understanding of flavor perception in beer by integrating sensory analysis with chemical analysis (HS‐SPME/GC‐MS), especially correlating sensory perceptions with specific volatile compounds. Sensory analysis revealed a preference for the passion fruit juice formulation, likely due to 18 compounds with medium to high flavor intensity. Notable among these are 6‐methylhept‐5‐en‐2‐one (citrus), (4R)‐1‐methyl‐4‐prop‐1‐en‐2‐ylcyclohexene (citrus), benzaldehyde (fruity), and 2‐methylpropyl hexanoate (fruity).

## Author Contributions

Marcos Edgar Herkenhoff: conceptualization, methodology, formal analysis, investigation, data curation, writing–original draft, and writing–review and editing. Ana Beatriz Praia: methodology, investigation, data curation, writing–original draft, and writing–review and editing. Guilherme Dilarri: data curation, writing–original draft, and writing–review and editing. Oliver Brödel: methodology, investigation, data curation, and writing–review and editing. Marcus Frohme: writing–review and editing and supervision. Susana Marta Isay Saad: conceptualization, writing–review and editing, supervision, project administration, and funding acquisition.

## Funding

This study was supported by the São Paulo Research Foundation (FAPESP, projects #2018/21584‐4, #2018/12190‐2, and #2013/07914‐8, and fellowships #2019/02583‐0 and #2021/08621‐0), Coordenação de Aperfeiçoamento de Pessoal de Nível Superior (CAPES: #88881.187323/2018‐01, #88882.376972‐2019‐01, #88887.473545/2020‐00), and Conselho Nacional de Desenvolvimento Científico e Tecnológico (CNPq: #308696/2022).

## Conflicts of Interest

The authors declare no conflicts of interest.

## Data Availability

The data that support the findings of this study are available from the corresponding author upon reasonable request.
